# Association of Graduate Medical Education With Hospital Performance and Patient Outcomes

**DOI:** 10.1001/jamanetworkopen.2020.34196

**Published:** 2021-01-28

**Authors:** Radoslav Zinoviev, Harlan M. Krumholz, Kevin Pirruccio, Howard Forman

**Affiliations:** 1 Department of Internal Medicine, Yale New Haven Hospital, New Haven, Connecticut; 2 now with Department of Cardiovascular Medicine, Heart, Vascular, and Thoracic Institute Cleveland Clinic, Cleveland, Ohio; 3 Center for Outcomes Research and Evaluation, Yale New Haven Hospital, New Haven, Connecticut; 4 Division of Cardiology, Department of Internal Medicine, Yale University School of Medicine, New Haven, Connecticut; 5 Department of Health Policy and Management, Yale School of Public Health, New Haven, Connecticut; 6 Perelman School of Medicine, University of Pennsylvania, Philadelphia; 7 Yale School of Management, New Haven, Connecticut; 8 Yale School of Public Health, New Haven, Connecticut; 9 Department of Radiology and Biomedical Imaging, Yale School of Medicine, New Haven, Connecticut

## Abstract

**Importance:**

Graduate medical education (GME) funding consists of more than $10 billion annual subsidies awarded to academic hospitals to offset the cost of resident training. Critics have questioned the utility of these subsidies and accountability of recipient hospitals.

**Objective:**

To determine the association of GME funding with hospital performance by examining 3 domains of hospital operations: financial standing, clinical outcomes, and resident academic performance.

**Design, Setting, and Participants:**

This study is an economic evaluation of all academic centers that received GME funding in 2017. GME funding data were acquired from the Hospital Compare Database. Statistical analysis was performed from May 2016 to April 2020.

**Exposures:**

GME funding.

**Main Outcomes and Measures:**

This study assessed the association between GME funding and each aspect of hospital operations. Publicly available hospital financial data were used to calculate a financial performance score from 0 to 100 for each hospital. Clinical outcomes were defined as 30-day mortality, readmission, and complication rates for a set of predefined conditions. Resident academic performance was determined by Board Certification Examination (BCE) pass rates at 0, 2, and 5 years after GME funding was awarded. Confounder-adjusted linear regression models were used to test association between GME funding data and a hospital’s financial standing, clinical outcomes, and resident academic performance.

**Results:**

The sample consisted of 1298 GME-funded hospitals, with a median (IQR) of 265 (168-415) beds and 32 (10-101) residents per training site. GME funding was negatively correlated with hospitals’ financial scores (β = –7.9; 95% CI, –10.9 to –4.8, *P* = .001). Each additional $1 million in GME funding was associated with lower 30-day mortality from myocardial infarction (–2.34%; 95% CI, –3.59% to –1.08%, *P* < .001), heart failure (–2.59%; 95% CI, –3.93% to –1.24%, *P* < .001), pneumonia (–2.20%; 95% CI, –3.99% to –0.40%, *P* = .02), chronic obstructive pulmonary disease ( –1.20%; 95% CI, –2.35% to –0.05%, *P* = .04), and stroke (–3.40%; 95% CI, –5.46% to –1.33%, *P* = .001). There was no association between GME funding and readmission rates. There was an association between higher GME funding and higher internal medicine BCE pass rates (0.066% [95% CI, 0.033% to 0.099%] per $1 million in GME funding; *P* < .001).

**Conclusions and Relevance:**

This study found a negative linear correlation between GME funding and patient mortality and a positive correlation between GME funding and resident BCE pass rates in adjusted regression models. The findings also suggest that hospitals that receive more GME funding are not more financially stable.

## Key Points

**Question:**

Is graduate medical education funding associated with hospital perfomance and patient outcomes?

**Findings:**

This economic evaluation of 1298 academic hospitals in the US assessed the association between confounder-adjusted graduate medical education funding and hospital financial standing, patient outcomes, and resident academic performance. A statistically significant association was found between higher graduate medical education funding and worse hospital financial standing, reduced patient mortality, and improved resident academic performance.

**Meaning:**

These findings suggest that graduate medical education funding does not improve the financial standing of hospitals but is associated with improved patient outcomes and resident academic performance.

## Teaser

This economic evaluation examines graduate medical education funding, an annual subsidy awarded to academic hospitals, and whether it is associated with a hospital’s financial standing, clinical outcomes, or resident academic performance.

## Introduction

Introduced in 1944, graduate medical education (GME) funding is a subsidy designed to offset the cost of resident training. It is primarily funded by the federal government as part of Medicare, Medicaid, the Veterans Health Administration, and other smaller sources.^1,2^ In 1983, GME funding was divided into Direct Medical Education funding, which reflects the size and costs of each resident training program and the share of Medicare patients at the hospital, and Indirect Medical Education funding to support the seemingly higher costs associated with resident training.^3,4^ In the years that followed, the mean GME subsidy per resident ($105 000)^5^ exceeded the mean resident salary ($55 987)^6^ and fringe benefits (estimated at 30%),^7^ resulting in a dramatic growth in the number of residents until the Balanced Budget Act of 1997 capped the number of Medicare-funded residency positions.^2^ The period of unrestricted growth resulted in an inequitable distribution of GME funding, with more funds given to specialty programs and with considerable geographic disparity that favors the northeastern US.^8^ Despite periodic attempts by the federal government to normalize GME payments,^1^ there is a 3-fold disparity between the GME dollars per resident allotted to the least- and most-funded states.^4^ The number of GME-sponsored resident positions across the US varies from 16.3 to 771.3 per million inhabitants, and in 2010, 68 sites received more than $200 000 GME dollars per resident.^4^ A 2013 study by the RAND Corporation^7^ estimated the cost of residency training at $135 000, far less than the value of residents’ work, estimated to be as much as $254 000 per year (this includes the mean GME subsidy per resident of $115 000).

With Medicare and Medicaid subsidies for GME surpassing $10 billion per year^5^ and reports of unequal distribution, critics have called into question the lack of accountability of recipient hospitals and have suggested that GME funding provides hospitals with excess revenue.^2^ In 2014, an Institutes of Medicine committee charged with investigating the governance and financing of GME^9^ published a report detailing a lack of transparency, inequitable distribution, and inability of GME funding to meet its goals. Although the committee did not find data regarding the outcomes of GME financing, they argued that decreasing GME funding could disrupt physician training and jeopardize patient care and proposed payment reforms that reward performance and ensure accountability. A 2020 *JAMA* viewpoint highlights the arguments questioning the utility of GME funding: that training costs and payer type are not as different at teaching hospitals as originally thought.^10^

Despite this considerable debate regarding the importance of GME funding, to our knowledge, there are no objective data on the role of GME funding in hospitals. The aim of this study is to explore what association, if any, may exist between GME dollars and the clinical and academic performance of residency training programs. Improved understanding of associations may guide the discussion on GME reform and future investigation in this area.

## Methods

The Yale University institutional review board deemed this study exempt because all databases provided deidentified patient data. According to Office for Human Research Protections guidelines, informed consent was not required because patient data was deidentified. This study followed the Consolidated Health Economic Evaluation Reporting Standards (CHEERS) reporting guideline.

We conducted secondary analysis on publicly available data on hospital finances, patient mortality rates, and internal medicine resident Board Certification Examination (BCE) pass rates, and used multivariable regression models to evaluate the association between GME funding and these outcomes. Databases provided deidentified patient data. All data are from fiscal year 2017, unless otherwise indicated. GME funding, number of residents, and number of beds per hospital were downloaded from the Center for Medicare & Medicaid Services (CMS) Hospital Cost Reports^5^ database. This study focuses on the $10.7 billion federal portion of GME funding (5-year range: $10.0-$11.7 billion), excluding funds awarded to Veterans Affairs hospitals. Total annual GME dollars is the sum of indirect and direct GME payments and was the primary independent variable of interest. For residency programs with multiple training sites, the GME funding used in the analysis is the sum of funds awarded across all training sites.

Financial standing was assessed according to a ranking model created in a complementary study.^11^ In this study, we created a singular composite score out of 100, named the Yale Hospital Financial Score (YHFS), which provides a financial rank for each hospital. We validated the model by showing (1) reproducibility on data excluded from the model’s creation, (2) close association to predefined reference standard credit rating scores of Moody’s (90.5%) and Standard and Poor’s (88.8%), and (3) ability to predict expected trends in nonfiscal data, such as hospital size and Medicare percentage. We concluded that the YHFS was a reliable composite score of hospital financial standing.

In assessing clinical outcomes, we used patient outcomes and internal medicine BCE pass rates as a surrogate for the efficacy of clinical and academic training in residency programs, respectively. Patient outcomes data were downloaded from the CMS Hospital Compare database.^12^ We used publicly available 30-day mortality rates for heart failure, acute myocardial infarction, pneumonia, stroke, and chronic obstructive pulmonary disease. We used readmission rates for the same conditions and hospitalwide readmission rates. Patient safety metrics used were hospital-associated pressure ulcers, pneumothorax, serious complications, or postoperative sepsis, bleeding, serious blood clots, kidney injury, or death.

Internal medicine resident BCE pass rate data were found on the website of the American Board of Internal Medicine.^13^ We compared BCE pass rates for residents undergoing examination in 2017 to GME funding awarded during the year of examination (2017), the year of matriculation (2014), and 5 years before examination (2012), thereby evaluating both the immediate and long-term associations of GME funding and resident academic performance.

We used linear multivariable regression models with Huber-White variance estimates^14,15^ on hospital-level data to assess the association between GME funding and hospital financial standing, patient outcomes, and resident BCE pass rates. For each regression, we performed exploratory analyses using scatterplots^16^ and lowess curves^17^ to assess the relationship between the factor (GME funding) and outcome variable. These distributions showed that log transformation of GME is linearly associated with hospital financial standing, whereas GME alone is linearly associated with clinical outcomes and resident BCE pass rates. Therefore, logarithmic transformation of GME was used in analyses involving hospital financial standing but not in analyses involving clinical outcomes or resident BCE pass rates. Normality and homoscedasticity of residuals were checked for each regression model.

Models of hospital financial standing and clinical outcomes were adjusted for confounding by the following hospital characteristics and community demographic metrics for each hospital’s zip code: type of hospital (acute care, children’s hospital), number of hospital beds, number of residents, percentage of Medicare patients, and demographic characteristics of the surrounding community (median age, gender [percentage male], race [percentage White], primary language [percentage English speaking], education [percentage high school graduates], median income, and poverty status) downloaded from the 2010 decennial US Census.^18^ Models assessing the association between GME funding and resident BCE pass rates were not adjusted for community demographic characteristics given that these were not considered confounders in the association of GME funding and academic performance.

We used 1-sided hypothesis testing and the null hypothesis that no association is present was rejected at *P* ≤ .05. Statistical significance in subgroup analyses was assessed with Holm-Bonferroni correction. All statistical analyses in this study were performed using Stata statistical data analysis software version 15 (StataCorp) from May 2016 to April 2020.

## Results

### Sample Characteristics

In 2017, 1298 hospitals reporting to CMS received GME funding and comprise the initial sample in this analysis. These hospitals had a median (IQR) of 265 (168-415) beds, 12 900 (7 800-21 200) annual discharges, and 32 (10-101) residents per training site. These hospitals received a median (IQR) of $2.64 ($0.49-$8.39) million per site, with a median (IQR) of $100 500 ($58 000-$134 700) per trainee. These hospital characteristics were positively skewed, with most hospitals falling below the mean for each category. Of these GME-funded hospitals, 1098 (84.6%) had sufficient data for inclusion in the outcomes analysis and 347 (26.7%) had BCE pass rate data.

### Hospital Financial Standing

**Figure 1.**
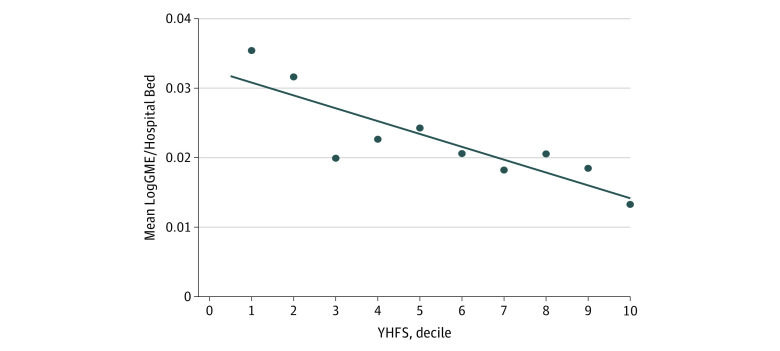
Mean Log–Graduate Medical Education (GME) Dollars Per Hospital Bed by Yale Hospital Financial Score (YHFS) Correlation between size-corrected LogGME funds and hospital financial ranking; shown per decile of the YHFS. For clarity, hospitals were grouped for each of 10 deciles from 0 to 100 of the YHFS, and mean LogGME was calculated for each group, shown as data points with trendline demonstrating decreasing YHFS with increasing LogGME/Hospital Bed.

In the 2017 data set, GME funds typically accounted for 1% to 2% of a hospital’s annual revenue. In a regression model of the association between GME funding and hospital revenue from patient care, each additional resident on staff was associated with $3.5 million in incremental revenue, even after adjusting for hospital size ($3.5 million; 95% CI, $1.0 to $6.0 million; *P* = .001). The amount of GME funding received by each hospital was not associated with an increase in revenue over and above the association with training program size ($1.9 million; 95% CI, –$12.6 to $16.4 million; *P* = .79). We found a negative linear correlation between log-transformed GME funding and overall financial standing (β = –7.9; 95% CI, –10.9 to –4.8; *P* = .001) even after adjusting for the aforementioned confounding variables (Figure 1).

We also performed analysis according to hospital types in our study. The mean financial score according to our model was 68.1 for children’s hospitals, 30.0 for critical access hospitals and 46.7 for general acute care hospitals. Accounting for hospital type reduced, but did not eliminate, the negative association of GME funding and hospital financial standing. Also, a hospital’s financial score was 0.25% lower for every additional percentage share of patients with Medicare, but adjusting for percentage Medicare patients did not eliminate the association with GME funding.

### Patient Outcomes Evaluation

**Figure 2.**
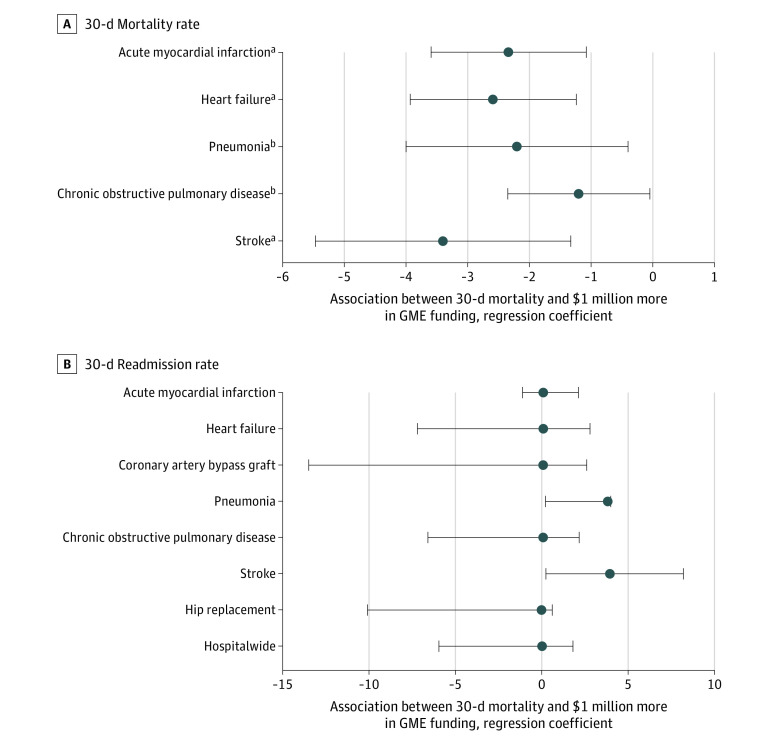
Association Between Graduate Medical Education (GME) Funding and 30-day Mortality Rates and 30-day Readmission Rates Multivariable linear regression models were used to evaluate the association between GME funding and 30-day mortality (A) and readmission from acute myocardial infarction, heart failure, pneumonia, chronic obstructive pulmonary disease, and stroke, with additional measures of coronary artery bypass graft, hip replacement, and hospitalwide for readmission rates (B). Regression coefficients are shown in hundreds of millions. Models were adjusted for number of hospital beds, number of residents, total number of hospital staff, percentage of Medicare patients, hospital financial standing, and community demographic characteristics: sex, race, language, primary language, education, median income, poverty status, and median age. ^a^*P* < .01. ^b^*P* < .05.

In assessing patient outcomes data, $1 million more in GME funding was associated with lower 30-day mortality rates for myocardial infarction (–2.34%; 95% CI, –3.59% to –1.08%; *P* < .001), heart failure (–2.59%; 95% CI, –3.93% to –1.24%; *P* < .001), pneumonia (–2.20%; 95% CI, –3.99% to –0.40%; *P* = .02), chronic obstructive pulmonary disease (–1.20%; 95% CI, –2.35% to –0.05%; *P* = .04), and stroke (–3.40%; 95% CI, –5.46% to –1.33%; *P* = .001) after adjusting for hospital and community demographic variables (Figure 2A). All 5 associations remained significant after Holm-Bonferroni correction for multiple tests of significance. There was no significant association between GME funding and 30-day readmission rates (Figure 2B) or patient safety metrics as defined by these criteria. The most important confounder in all models was the size of the hospital, represented as the number of hospital beds, which alone was associated with 42% of the variation in GME funding awarded to that hospital in each year (*r*^2^ = 0.42). Hospital size and the size of the residency program, expressed as the number of interns and residents per hospital, were associated with 79% of the variation in GME funding (*r*^2^ = 0.79).

### Resident Academic Performance

Finally, we examined the association between GME funding and resident education as measured by the pass rates for BCEs in internal medicine. We found a significant association between internal medicine BCE pass rates and GME funding awarded 5 years prior to taking the BCE (0.056% [95% CI, 0.023% to 0.089%] per $1 million in GME funding; *P* = .001), in the year of matriculation (0.064% [95% CI, 0.027% to 0.100%] per $1 million in GME funding; *P* = .001) and in the expected year of graduation (0.066% [95% CI, 0.033% to 0.099%] per $1 million in GME funding; *P* < .001).

## Discussion

This study found that hospitals with larger residency programs reported higher revenues, but the amount of GME funding was negatively correlated with a hospital’s financial standing. Conversely, our findings suggest a negative association between GME funding and 30-day patient mortality from several common life-threatening conditions. Similarly, there was a positive correlation between GME funding and resident performance on BCEs in internal medicine. These findings respond to growing criticism regarding the utility of GME funding.

We found that hospitals with larger programs earned higher revenues, but there was no apparent correlation with profitability. It is possible that the expenses of academic hospitals, among them the cost of training of residents, exceed the revenue benefit. Because financial standing is more complex than can be accounted for by these metrics, we found the need to expand our analysis beyond revenues and profitability. We examined many financial ratios, which are the simplest and most commonly used method for financial analysis but found that their data are limited to discrete aspects of operations. This motivated a parallel study in which we proposed a statistically based model for rating hospital financial standing. Using this model, we found that hospitals receiving more GME funding had lower financial standing, suggesting that GME funding may not increase the financial stability of a hospital as previously suggested. Many of the most financially stable hospitals are children’s hospitals, which receive reduced GME funding^19^ but have significant endowments from charitable donations.^5^ In fact, according to our model, children's hospitals were more financially stable than critical access and acute care hospitals, although accounting for hospital type did not eliminate the negative association of GME funding and hospital financial standing. Similarly, hospitals with a higher share of Medicare patients had lower financial scores, but adjusting for proportion of Medicare patients did not eliminate the negative association between GME funding and YHFS. It appears that hospitals with better financial standing receive less GME funding, which may not be necessary, nor does it appear to be sufficient for a hospital to become financially successful.

We proposed that patient outcomes data reflected the aptitude of clinical reasoning developed during residency, whereas resident performance on BCEs is suggestive of knowledge acquisition. We created confounder-controlled models and found that GME funding was associated with lower risk-standardized 30-day mortality rates after myocardial infarction, heart failure, pneumonia, stroke, and chronic obstructive pulmonary disease admission. The results were significant after accounting for a comprehensive set of confounder variables. We did not find significant associations between GME funding and readmission rates or Hospital Consumer Assessment of Healthcare Providers and Systems scores. Interestingly, the number of residents was positively correlated with 30-day mortality rates, but when the number of residents is held constant, additional GME funding was associated with improved outcomes. This suggests that increasing residency program size while keeping faculty size and GME funding constant (ie, disproportionate growth) may have a negative impact on patient outcomes. Alternatively, this may hint at differences between residency programs that could not be accounted for in this analysis. Future research should assess differences in GME payment and outcomes across program types (eg, primary care vs specialty training), which was outside the scope of this study.

We used resident BCE pass rates as a reflection of knowledge acquisition and found a positive association between GME funding and resident BCE pass rates in internal medicine awarded 0, 2, and 5 years prior to the examination date. Unfortunately, the lack of transparency in both the allocation of GME funds and internal operations of residency programs hinders in-depth analysis of specific differences between these programs. To our knowledge, this is the first published association between GME funding and academic training. Although they do not show causation, our data contradict claims that GME funding has no impact on resident education.

### Limitations

There are several limitations to this analysis. Our study is an economic evaluation, which limits our ability to make causal inferences. Nevertheless, the association that we observed is robust. The hospitals with more GME funding and better financial status had lower mortality and better resident performance. A further limitation is that patient mortality and readmissions data are available for only a few conditions and restrict the evaluation to only 30 days. GME payments are influenced by fees billed to Medicare, and procedure volume may be a confounder that is difficult to account for and is an important consideration for future work. Furthermore, it is impossible to extract the impact of residents vs other team members on patient outcomes. We cannot make assertions on the way that GME funding impacts residents’ clinical skills, many of which are shaped by an informal curriculum that cannot be standardized. Although BCE pass rates were the only publicly available proxy for program quality, they may not fully reflect the capacity of a resident to perform as a doctor. Our model does not account for factors not available to the public, such as intrinsic program characteristics or the caliber of residents on matriculation, which may influence BCE pass rates, or hospital renown or reputed quality, which may influence patient outcomes. Nevertheless, we believe that BCE pass rates at the end of residency training reflect each program’s ability to teach clinical knowledge in an impactful way. Further work is needed to show in what way and to what extent GME funding impacts patient outcomes and resident academic performance.

## Conclusions

GME funding has become an important topic in the discussion of health care reform. Its utility and efficacy have been called into question, while hospitals claim that they bear an increasing cost of resident training. We examined the association between GME funding and hospital finances, clinical outcomes, and resident training. We found that GME funding is negatively correlated with the financial standing of hospitals, suggesting that these hospitals may be less financially resilient either because of the cost of resident training, the populations they serve, or other associated factors. Conversely, we found a significant association between GME funding and patient outcomes, a possible reflection of the clinical training of residents. The subsidy is also correlated with resident performance on internal medicine BCEs, suggesting that residents in better funded programs acquire more medical knowledge during their training. To our knowledge, this is the only published study to investigate the association between GME funding and hospital outcomes, and the first time that an association between the 2 has been shown. However, our data concurred that there are inequalities in the way that GME funding is distributed. There is also a lack of clarity in the way that GME funds are allocated by each hospital, highlighting the importance of the recommendations of the Institutes of Medicine committee’s 2014 report to implement a “transparent, performance-based system”^9^ for the governance of GME. On the basis of these data, GME funding may be an important factor in the training of future doctors, although we strongly echo the increasing call for transparent use and allocation reform.
