# Errors in Figure 2B

**DOI:** 10.1001/jamanetworkopen.2021.1827

**Published:** 2021-02-25

In the Original Investigation titled “Association of Graduate Medical Education With Hospital Performance and Patient Outcomes,”^1^ published January 28, 2021, there were errors in the x-axis label and the data markers for regression coefficients and 95% CIs in panel B of Figure 2. The x-axis label should have been “Association between 30-d readmission rate and $1 million more in GME funding, regression coefficient.” In addition, the data markers for acute myocardial infarction, heart failure, coronary artery bypass graft, pneumonia, chronic obstructive pulmonary disease, stroke, hip replacement, and hospitalwide were positioned incorrectly. This article has been corrected.^1^
